# The impact of pharmacist-led medication therapy management on the efficacy of cancer pain control: a pre-post interventional study

**DOI:** 10.1186/s40780-026-00544-8

**Published:** 2026-01-27

**Authors:** Yu Dong, Yufeng Liu, Shunfu Zheng, Li Feng

**Affiliations:** Department of Pharmcacy, People’s Hospital of Kaihua, Quzhou, Zhejiang China

**Keywords:** Cancer pain management, Medication therapy management (MTM), Drug-related problems (DRPs), Pharmacist intervention, Numerical rating scale (NRS)

## Abstract

**Background:**

Cancer pain remains a prevalent and debilitating symptom among patients with advanced malignancies, significantly compromising quality of life. While clinical guidelines for pain management exist, real-world challenges such as polypharmacy, poor adherence, and drug-related problems (DRPs) hinder effective treatment. Pharmacist-led Medication Therapy Management (MTM) offers a structured approach to address these challenges, but its role in cancer pain management remains underexplored.

**Aim:**

This study aimed to evaluate the effect of pharmacist-led MTM on cancer pain management.

**Methods:**

A pre-post interventional study was conducted using the MTM services implemented in January 2023.

**Results:**

A total of 246 patients were included in the pre-MTM group (*n* = 100) and MTM (*n* = 146) groups. Pharmacist-led MTM was associated with better cancer pain score at 30-day follow-up (3.85 ± 1.07 VS 4.85 ± 1.47, *P* < 0.001) and greater reduction in pain scores (1.92 ± 0.84 VS 2.81 ± 1.21, *P* < 0.001) compared to the pre-MTM group. In Additional, the MTM effectively identified and resolved the DRPs, achieving a resolution rate of 73.81%. A lower incidence of adverse drug reactions, including constipation, nausea, and vomiting, was observed in the MTM group (*P* < 0.05). Furthermore, pharmacist-led MTM was associated with a higher proportion of patients with high medication adherence, with 67.81% in the MTM group compared with 45% in the pre-MTM group (*P* < 0.001). Pharmacist-led MTM was associated with higher patient satisfaction with treatment (4.71 ± 1.14 vs. 3.04 ± 2.07, *P* < 0.001) and follow-up (3.92 ± 1.76 vs. 3.46 ± 1.70, *P* = 0.02) compared with the pre-MTM group.

**Conclusion:**

Pharmacist-led MTM plays a crucial role in optimizing cancer pain management by reducing DRPs, improving adherence, minimizing adverse drug reactions, and enhancing patient satisfaction. It is an essential component of comprehensive, individualized care that ultimately improves the clinical outcomes and quality of life of patients with cancer.

**Supplementary Information:**

The online version contains supplementary material available at 10.1186/s40780-026-00544-8.

## Introduction

Cancer pain is a prevalent and distressing symptom among patients with advanced malignancies, affecting 70–90% of patients with at some stage of their illness [1, 2]. Inadequately managed pain significantly compromises physical, psychological, and social well-being [3]. Although various clinical guidelines emphasize standardized pain treatment, disparities persist in real-world practice due to polypharmacy, comorbidities, and suboptimal adherence to therapeutic regimens.

Pharmacists are integral members of multidisciplinary healthcare teams and play a vital role in promoting rational drug use. In recent years, multiple studies have explored the impact of pharmacist interventions on cancer pain management. For instance, Shrestha et al. [4] demonstrated the feasibility and preliminary effectiveness of integrating clinical pharmacists into cancer pain management teams in Nepal. Liu et al. [5] and Zhang et al. [6] reported that pharmacist involvement was effective in resolving drug-related problems (DRPs) and reducing medication costs. Zheng et al. [7] further showed that pharmacist-led opioid management programs improved medication adherence and patient-reported outcomes such as quality of life. However, these studies varied widely in their intervention strategies and outcome indicators.

To address these challenges, pharmacist-led Medication Therapy Management (MTM) offers a structured and standardized model of pharmaceutical care. MTM encompasses a range of professional services provided by pharmacists with expertise in pharmacology, including patient education, medication counseling, and adherence support. These services aim to enhance treatment efficacy by improving medication adherence, preventing medication errors, and empowering patients in self-medication management [8–10]. MTM has been widely implemented in chronic disease management, such as diabetes [11] and hypertension [8]. However, limited data specifically evaluates the impact of MTM as a standardized service in the context of cancer pain management.

This study aimed to evaluate the application of Pharmacist-led MTM in patients with cancer pain to ensure comprehensive pharmaceutical care throughout treatment. This study assessed key clinical outcomes, including pain relief rates, incidence of adverse drug reactions, and medication adherence.

## Materials and methods

### Ethical approval

The study protocol was approved by the Ethics Review Committee of the People’s Hospital of Kaihua on September 23, 2022 (Approval No.: 2022-03) and strictly adhered to the ethical guidelines of the Declaration of Helsinki.

### Inclusion and exclusion criteria

Patients were eligible for inclusion if they met the following criteria: (1) age ≥ 18 years, (2) pathologically or cytologically confirmed advanced malignant tumors, (3) receiving opioid analgesic therapy, (4) expected survival time ≥ 1 month, and (5) conscious and able to cooperate with questionnaire surveys.

Patients were excluded if they met any of the following criteria: (1) no cancer pain, and (2) impaired consciousness or mental disorders preventing effective communication.

### Data sources

This study included patients with cancer pain admitted to our hospital between January 2022 and December 2024. Pharmacist-led MTM services are expected to be introduced in January 2023. Patients admitted between January 2022 and December 2022 were assigned to the pre-MTM group, where those admitted between January 2023 and December 2024 were assigned to the MTM group. All procedures in this study were conducted in accordance with the ethical standards of the responsible committee on human experimentation (institutional and national) and the 1975 Helsinki Declaration (revised in 2000).

The Ethics Committee of Kaihua People’s Hospital approved the use of patient data, materials, and/or test results for research purposes (2022-03).

### Intervention measures

Pre-MTM Group: Patients received standard treatment provided by clinical physicians and nursing staff following the 2018 Cancer Pain Treatment Guidelines [12].

MTM Group: In addition to standard treatment, pain clinical pharmacists provided MTM services and pharmaceutical interventions.

### MTM services and pharmaceutical interventions

#### MTM services

Clinical pharmacists assessed patients with cancer pain following the principles of standardized, quantitative, comprehensive, and dynamic evaluation, as outlined in the 2018 Cancer Pain Treatment Guideline [12]. A personalized medication plan was developed in collaboration with clinicians, based on each patient’s condition and pain status. At discharge, the patient received comprehensive medication counseling. Pharmacists conduct weekly follow-ups via phone or in person for 30 days post-discharge to assess: (1) pain levels; (2) adverse drug reactions; (3) acute pain episodes; (4) medication use and adherence. Medication plans were adjusted as needed in consultation with physicians.

#### Pharmaceutical interventions

During hospitalization, the pharmaceutical care network europe (PCNE) classification system (version 9.0) was used to identify and address the DRPs in the MTM group. The classification system includes five key components: (1) problem (P): number and type of identified DRPs; (2) cause (C): underlying causes of DRPs; (3) planned interventions (I): types of interventions implemented; (4) intervention acceptance (A): acceptance rate of pharmacist interventions; and (5) outcome (O): effectiveness of the interventions.

Each component was analyzed in detail, and interventions were categorized into three levels: (1) patient level: providing medication education, drug counseling, and adherence monitoring to enhance self-management; and (2) physician level, collaborating with physicians on drug selection and optimizing medication therapy to resolve actual or potential DRPs. (3) Medication level: Adjusting the dosage, administration schedules, and other drug-related parameters to improve efficacy and safety.

### Observational indicators

#### DRPs

The PCNE classification system was used to analyze the types, causes, intervention measures, and outcomes of DRPs in the MTM group during hospitalization.

#### Pain assessment

Pain was assessed in both groups using the following: (1) numerical rating scale (NRS) scores at the first and 30-day follow-up, and (2) incidence of breakthrough pain episodes (mean number of breakthrough pain episodes during the 30-day follow-up period).

#### Incidence of adverse reactions

The incidence rates of constipation, nausea, vomiting, and other adverse reactions were recorded at the first visit and 30-day follow-up in both groups.

#### Adherence

Medication adherence in both groups was assessed on the second visit using the Chinese version of the Morisky Medication Adherence Scale (MMAS-8). Permission to apply for the MMAS-8 score was obtained. The total score was eight, with scores classified as follows: (1) low adherence (< 6), (2) moderate adherence (6–7), and (3) high adherence (7–8).

A hospital-made treatment satisfaction survey (Table S1) and follow-up satisfaction survey (Table S2) were conducted. Each satisfaction questionnaire consisted of six items, each rated on a 3-point Likert scale (0 = dissatisfied, 0.5 = neutral, 1 = satisfied), yielding a total score ranging from 0 to 6, with higher scores indicating greater satisfaction.

**Table 1:** Socio-demographic characteristics of cancer pain patients (*n* = 246)

		pre-MTM group (*n* = 100)	MTM group (*n* = 146)	*P* value
Age		67.98 ± 10.09	67.67 ± 11.04	0.412
Gender				
	Male	67	100	0.805
	Female	33	46	
Marital status				
	Married	76	116	0.521
	Single/Divorced/Unknown	24	30	
Race				
	Han	98	142	0.712
	other	2	4	
BMI		21.96 ± 2.86	21.50 ± 3.38	0.144
Diagnosis				0.865
	lung cancer	34	49	
	liver cancer	18	24	
	colorectal cancer	12	15	
	gastric cancer	5	14	
	esophagus cancer	7	10	
	other	24	34	
Cancer stage				
	III	32	47	0.998
	IV	64	93	
	Unknown	4	6	
Health ensurance coverage				0.824
	Yes	86	127	
	No	14	19	
Baseline opioid dosage (mg oral morphine/day)				0.422
		47.92 ± 39.32	47.05 ± 34.62	
Pain type				0.662
	Somatic pain	52	81	
	Visceral pain	24	28	
	Neuropathic pain	24	37	
Hospital duration		7.20 ± 8.15	7.27 ± 7.01	0.470

**Table 2:** Control rate of cancer pain

	pre-MTM group (*n* = 100)	MTM group (*n* = 146)	*P* value
cancer pain score at first visit	6.81 ± 1.85	6.66 ± 1.89	0.266
cancer pain score at 30-day follow-up	4.85 ± 1.47	3.85 ± 1.07	*P* < 0.001
changes in cancer pain score	1.92 ± 0.84	2.81 ± 1.21	*P* < 0.001
incidence of breakthrough pain*	2.65 ± 1.26	1.75 ± 1.26	*P* < 0.001

*mean number of breakthrough pain episodes during the 30-day follow-up period

**Table 3:** Identified problems and resolved problem in the MTM group according to the PCNE^a^ DRP^b^ classification tool

Primary Domain	Problem	Cases found	%	Cases solved	%
P1 Treatment effectiveness	P1.1 No effect of drug treatment	5	5.95	5	100.00
	P1.2 Effect of drug treatment not optimal	18	21.43	18	100.00
	P1.3 Untreated symptoms or indication	6	7.14	6	100.00
	P1.4 Wrong effect of drug administered	3	3.57	3	100.00
P2 Treatment safety	P2.1 Adverse drug event (possibly) occurring	31	36.90	15	48.39
	P2.2 Toxic effect suspected	3	3.57	3	100.00
	P2.3 Side effect suffered	0	0.00	0	/
	P2.4 Contraindicated drug prescribed	0	0.00	0	/
P3 Other	P3.1 Problem with cost-effectiveness	0	0.00	0	/
	P3.2 Patient dissatisfied with therapy	18	21.43	12	66.67
	P3.3 Unclear problem / miscellaneous	0	0.00	0	/

^a^ Pharmaceutical Care Network Europe, ^b^ drug-related problem

**Table 4:** Identified causes in the intervention group based on the PCNE ^a^ DRP ^b^ classification tool

Primary Domain	Cause	Cases	%
C1 Drug selection	C1.1 Inappropriate drug according to guidelines/formulary	6	7.14
	C1.2 Inappropriate drug (within guidelines but otherwise contra-indicated)	5	5.95
	C1.3 No indication for drug	3	3.57
	C1.4 Inappropriate combination of drugs, or drugs and herbal medications, or drugs and dietary supplements	0	0.00
	C1.5 Inappropriate duplication of therapeutic group or active ingredient	0	0.00
	C1.6 No or incomplete drug treatment in spite of existing indication	0	0.00
	C1.7 Too many drugs prescribed for indication	0	0.00
C2 Drug form	C2.1 Inappropriate drug form (for this patient)	3	3.57
	C2.2 Inappropriate dose form	2	2.38
C3 Dose selection	C3.1 Drug dose too low	13	15.48
	C3.2 Drug dose too high	6	7.14
	C3.3 Dosage regimen not frequent enough	1	1.19
	C3.4 Dosage regimen too frequent	1	1.19
	C3.5 Dose timing instructions wrong, unclear or missing	0	0.00
C4 Treatment duration	C4.1 Duration of treatment too short	6	7.14
	C4.2 Drug dose too high	2	2.38
C5 Dispensing	C5.1 Prescribed drug not available	0	0.00
	C5.2 Necessary information not provided	0	0.00
	C5.3 Wrong drug, strength or dosage advised (OTC)	0	0.00
	C5.4 Wrong drug or strength dispensed	0	0.00
C6 Drug use process	C6.1 Inappropriate timing of administration or dosing intervals	6	7.14
	C6.2 Drug under-administered	4	4.76
	C6.3 Drug over-administered	4	4.76
	C6.4 Drug not administered at all	4	4.76
	C6.5 Wrong drug administered	0	0.00
	C6.6 Drug administered via wrong route	0	0.00
C7 Patient related	C7.1 Patient uses/takes less drug than prescribed or does not take the drug at all	16	19.05
	C7.2 Patient uses/takes more drug than prescribed	2	2.38
	C7.3 Patient abuses drug (unregulated overuse)	0	0.00
	C7.4 Patient uses unnecessary drugs	0	0.00
	C7.5 Patient takes food that interacts	0	0.00
	C7.6 Patient stores drugs inappropriately	0	0.00
	C7.7 Inappropriate timing or dosing intervals	0	0.00
	C7.8 Patient administers/uses the drug in a wrong way	0	0.00
	C7.9 Patient unable to use drug/form as directed	0	0.00
	C7.10 Inappropriate drug/therapeutic monitoring (incl. TDM)	0	0.00
C8 Patient transfer related	C8.1 Incomplete or incorrect transfer of medication information	0	0.00
	C8.2 Incomplete or incorrect transfer of patient information	0	0.00
	C8.3 No obvious cause	0	0.00
C9 Other	C9.1 Other cause; specify	0	0.00
	C9.2 No obvious cause	0	0.00

**Table 5:** Incidence of adverse reactions

	pre-MTM group *n* (%)	MTM group *n* (%)	*P* value
Constipation	30(30.0%)	23(15.8%)	0.008
Nausea	34(34.0%)	33(22.6%)	0.049
Vomiting	12(12.0%)	6(4.1%)	0.020
Other	5(5.0%)	3(2.1%)	0.201

**Table 6:** Adherence of cancer pain patients to treatment (*n* = 246)

	Pre-MTM group	MTM group	P value
	n (%)	n (%)	
Adherence-Low	19(19.0%)	6(4.1%)	*P* < 0.001
Adherence-Moderate	36(36.0%)	41(28.1%)	
Adherence-High	45(45.0%)	99(67.8%)	

**Table 7:** Satisfaction of treatment and follow-up

	Pre-MTM group (*n* = 100)	MTM group (*n* = 146)	*P* value
satisfaction of treatment	3.04 ± 2.07	4.71 ± 1.14	*P* < 0.001
satisfaction of follow-up	3.46 ± 1.70	3.92 ± 1.76	*P* = 0.02

### Statistical analysis

Statistical analyses were performed using SPSS version 24.0. Continuous data were expressed as mean ± standard deviation (x̄ ± s) and analyzed using the t-test. Categorical data are expressed as percentages (%) and were analyzed using the χ² test. Statistical significance was set at *P* < 0.05.

## Results

### General information of patients

In total, 246 patients met the inclusion criteria, with 100 in the pre-MTM group and 146 in the MTM groups. The baseline characteristics of the two groups are presented in Table 1. There were no statistically significant differences between the two groups in terms of age, sex, marital status, race, BMI, diagnosis, cancer stage, or health insurance coverage (*P* > 0.05). The mean baseline opioid dosage, expressed as oral morphine equivalents, did not differ significantly between the pre-MTM group and the MTM group (47.92 ± 39.32 mg/day vs. 47.05 ± 34.62 mg/day, *P* = 0.422). Regarding pain type distribution, no statistically significant differences were observed between the groups (*P* = 0.662). In the pre-MTM group, 52% of patients had somatic pain, 24% had visceral pain, and 24% had neuropathic pain, compared with 55.5% (81/146), 19.2% (28/146), and 25.3% (37/146), respectively, in the MTM group. The hospitalization duration was comparable between the two groups (pre-MTM: 7.20 ± 8.15 days; MTM: 7.27 ± 7.01 days; *P* = 0.470; Table 1).

### Control rate of cancer pain

The impact of pharmacist-led MTM on the control rate of cancer pain is shown in Table 2. At baseline, there was no significant difference in the initial cancer pain scores between the pre-MTM group (6.81 ± 1.85) and MTM group (6.66 ± 1.89) (*P* = 0.266) as shown in Table 2. At the 30-day follow-up, the pain score in the MTM group had decreased by 2.81 ± 1.21 points, which was significantly greater than the reduction in the pre-MTM group (1.92 ± 0.84 points, *P* < 0.001). The pain score at the 30-day follow-up in the MTM group (3.85 ± 1.07) was significantly lower than that in the pre-MTM group (4.85 ± 1.47; *P* < 0.001). During the 30-day follow-up, the MTM group had a significantly lower mean number of breakthrough pain episodes than the pre-MTM group (1.75 ± 1.26 vs. 2.65 ± 1.26, *P* < 0.001). During the 30-day follow-up, the MTM group was associated with lower pain scores and fewer breakthrough pain episodes compared with the pre-MTM group.

### DRPs and resolution in the MTM group

The impact of pharmacist-led MTM on DRP and resolution is shown in Table 3. Among the 146 patients in the MTM group, 84 DRPs were identified, with an average of 0.58 DRPs per patient. A total of three primary problem domains (P1–P3) were identified in the MTM group according to the PCNE DRP classification tool (Table 3). Most DRPs were related to “treatment safety” (P2, 40.48%) and “treatment effectiveness” (P1, 38.09%), followed by a smaller proportion classified as “other problems” (P3, 21.43%). Within the domain of “treatment effectiveness” (P1), the most frequent issues were “suboptimal treatment effect” (P1.2, 21.43%), “untreated symptoms or indications” (P1.3, 7.14%), and “no effect of drug treatment” (P1.1, 5.95%). All P1-category problems were successfully resolved (100%). For “treatment safety” (P2), the predominant problem was “adverse drug events possibly occurring” (P2.1), accounting for 36.90% of all DRPs; however, only 48.39% of these were fully resolved. “Toxic effects suspected” (P2.2) accounted for 3.57%, all of which were resolved. No cases were recorded for P2.3 or P2.4. Within the “other” (P3) domain, “patient dissatisfaction with therapy” (P3.2) constituted 21.43% of all DRPs, with a resolution rate of 66.67%.

As shown in Table 4, based on the PCNE DRP classification, the most frequent causes of DRPs in the MTM group were associated with dose selection (C3, 27.98%), particularly drug dose too low (C3.1, 15.48%) and drug dose too high (C3.2, 7.14%). This was followed by drug use process issues (C6, 23.81%), including inappropriate timing of administration, under-administration, over-administration, and drugs not administered at all. Patient-related factors (C7, 21.43%) were also common, with patients taking less medication than prescribed or not taking it at all (C7.1, 19.05%) as the leading cause. Additional causes were identified within drug selection (C1, 16.67%) and drug form (C2, 5.95%), whereas no causes were found in the dispensing (C5), patient transfer (C8), or other categories (C9).

### Occurrence of adverse reactions

The impact of pharmacist-led MTM on the occurrence of adverse reaction in 30-day follow-up is shown in Table 5. In the pre-MTM group, constipation occurred in 30 patients (30.0%), nausea in 34 (34.00%), vomiting in 12 (12.00%), and other adverse reactions in 5 (5.00%). In the MTM group, constipation occurred in 23 patients (15.75%), nausea in 33 (22.60%), vomiting in 6 (4.11%), and other adverse reactions in 3 (2.05%). The incidence of constipation, nausea, and vomiting were significantly lower in the MTM group than in the pre-MTM group (*P* < 0.05). During the 30-day follow-up, pharmacist-led MTM was associated with lower incidences of drug reactions, such as constipation, nausea, and vomiting compared with the pre-MTM group.

### Medication adherence

The interval to the second outpatient visit showed no significant difference (pre-MTM: 14.33 ± 8.70 days; MTM: 15.59 ± 7.31 days; *P* = 0.111). These findings indicate that differences in follow-up timing were minimal and unlikely to influence the assessment of medication adherence. The impact of pharmacist-led MTM on medication adherence is shown in Table 6. Medication adherence in patients with cancer pain was assessed. In the pre-MTM group, 19.00%, 36.00%, and 45.00% were Adherence-low, Adherence-moderate, and Adherence-high, respectively. In the MTM group, 4.11%, 28.08%, and 67.81% in the Adherence-Low, Adherence-Moderate, and Adherence-High groups, respectively. Compared to the pre-MTM group, the proportion of adherence-high patients in the MTM group significantly increased, while the proportion of adherence-low patients also decreased. Pharmacist-led MTM was associated with a higher proportion of patients with high medication adherence and a lower proportion of patients with low adherence compared with the pre-MTM group.

### Satisfaction of treatment and follow-up

The association between pharmacist-led MTM and patient satisfaction with treatment and follow-up is shown in Table 7. Satisfaction with treatment and follow-up in the MTM group was 4.71 ± 1.14 and 3.92 ± 1.76, respectively, which was higher than that observed in the pre-MTM group (satisfaction with treatment: 3.04 ± 2.07, *P* < 0.001; satisfaction with follow-up: 3.46 ± 1.70, *P* = 0.02).

## Discussion

Cancer pain is a common and debilitating symptom that significantly impairs patients’ quality of life and overall well-being [13–15]. Given the complexity of cancer pain regimens, including opioid therapy, polypharmacy, and adverse drug reactions (ADRs) risk, comprehensive and individualized management is essential for optimizing outcomes [14, 16]. This study provides real-world evidence that pharmacist-led MTM is associated with favorable outcomes in the management of cancer pain. By implementing a structured pharmaceutical care model, MTM was associated with favorable outcomes across multiple clinical domains, including better pain control, higher medication adherence, fewer DRPs, and a lower incidence of ADRs.

The findings of this study underscore the significance of pharmacists’ involvement in cancer pain management. Unlike general pharmacist interventions, which often involve isolated or reactive responses to specific issues [5–7, 17], MTM represents a formalized and systematic approach to pharmaceutical care [8, 11]. In our study, pharmacist-led MTM was associated with better pain control (Table 2), fewer DRPs (Table 3), lower incidence of ADRs (Table 5), better adherence (Table 6) and patient satisfaction (Table 7). It seemed that pharmacist-led MTM has more consistent outcomes compared to fragmented or ad hoc pharmacist interventions reported in previous studies [6, 15, 18, 19].

During the 30-day follow-up, the MTM group was associated with lower pain scores and fewer breakthrough pain episodes compared with the pre-MTM group (Table 2). Importantly, patients in the MTM group also experienced a greater reduction in pain scores from baseline, suggesting improved dynamic pain control rather than merely lower absolute scores at follow-up. These findings are consistent with previous studies indicating that pharmacist involvement facilitates more individualized pain assessment and opioid titration, ultimately contributing to better symptom control [7, 20, 21]. The regular monitoring and follow-up inherent in MTM likely contributed to more timely adjustments in analgesic regimens, aligning with best practices for dynamic cancer pain management.

Pharmacist-led MTM was associated with favorable outcomes in the identification and resolution of DRPs. In this study, 146 patients in the MTM group, 84 DRPs were identified, and over 73% were successfully resolved. This intervention success rate is consistent with prior research from Liu et al. [5] and Zhang et al. [6], who also highlighted the pharmacist’s ability to identify medication-related issues early and to intervene effectively. In our study, the resolution rates for adverse reactions and patient dissatisfaction with therapy were lower compared to other DRP categories (Table 3). ADR-related DRPs had lower resolution rates because many opioid-related ADRs (e.g., constipation, nausea) are intrinsic to opioid therapy and may not be fully reversible despite optimization. This relatively lower resolution rate of patient dissatisfaction with therapy may be attributed to the multifactorial nature of patient dissatisfaction, which is often influenced by patients’ expectations, beliefs, prior treatment experiences, and psychological factors [22, 23]. Unlike medication-related or safety-related DRPs, dissatisfaction with therapy typically requires sustained communication, trust-building, and behavioral reinforcement over time, and therefore may not be fully resolved during a single hospitalization or short-term follow-up period [24, 25].

Medication adherence was higher in the MTM group, with a greater proportion of patients achieving high adherence compared with the pre-MTM group. These findings are in line with previous evidence indicating that pharmacist-led interventions may play an important role in supporting medication adherence among cancer patients [18].

Pharmacists in the MTM model likely contributed through reinforcement of medication instructions, addressing misconceptions about opioids, and enhancing patient empowerment [17].

Additionally, the MTM group had lower incidence of ADRs such as constipation, nausea, and vomiting. This observation is consistent with previous studies suggesting that pharmacist involvement may facilitate earlier identification and improved management of ADRs, which may contribute to a lower reported incidence [6, 18].

Pharmacist-led MTM was associated with higher patient satisfaction with treatment and follow-up (Table 7). This high level of satisfaction reflects the positive impact of MTM on both physical and psychological aspects of cancer pain management. Satisfaction is an important patient-reported outcome that correlates not only with trust and communication but also with adherence and clinical response [4, 20].

This study has several limitations that should be acknowledged. First, the pre–post observational design without randomization may introduce unmeasured confounding, thereby limiting the strength of causal inference regarding the intervention’s effectiveness. Second, baseline medication adherence was not assessed in the pre-MTM group, preventing a difference-in-differences analysis and restricting the interpretation of adherence-related outcomes. Third, DRPs were systematically documented only in the MTM group; thus, direct comparison of DRPs between groups was not possible. Fourth, several outcome measures—including satisfaction and adherence—relied on patient self-report, which may be subject to recall or reporting bias. Finally, this was a single-center study conducted in a tertiary hospital, which may limit the generalizability of the findings to other clinical settings. Future multicenter, randomized controlled studies are warranted to confirm these results and strengthen the evidence base for pharmacist-led MTM in cancer pain management.

In conclusion, the results of this study demonstrated that pharmacist-led MTM is associated with favorable outcomes in cancer pain management. MTM may play an important role in optimizing cancer pain management by addressing DRPs, enhancing medication adherence, reducing ADRs, and improving patient satisfaction. Our study underscores the value of formalized MTM services in delivering consistent, comprehensive pharmaceutical care. These results support broader implementation of MTM in oncology settings and provide a basis for future multicenter or long-term studies to validate and extend our findings.

## Electronic Supplementary Material

Below is the link to the electronic supplementary material.

Supplementary Table S1: Pain Treatment Satisfaction Questionnaire

Supplementary Table S2: Cancer Pain Treatment Follow-up Satisfaction Questionnaire

## Data Availability

The data that support the findings of this study are available from the corresponding author, Dong Yu, upon reasonable request.

## Abbreviations

MTM: Medication therapy management
DRPs: Drug-related problems
PCNE: Pharmaceutical care network Europe
MMAS-8: Morisky Medication Adherence Scale
