# Supplementary material for: The impact of pharmacist-led medication therapy management on the efficacy of cancer pain control: a pre-post interventional study
Source: J Pharm Health Care Sci. 2026 Jan 27;12:24. doi: 10.1186/s40780-026-00544-8 (PMC12918049; doi:10.1186/s40780-026-00544-8)
Supplement: Supplementary file 1 — Supplementary Table S1: Pain Treatment Satisfaction Questionnaire [file 40780_2026_544_MOESM1_ESM.docx]

**Table S1 Pain Treatment Satisfaction Questionnaire**

6 items, 3-point scale, total score 0–6

**Instructions**

This questionnaire is used to evaluate the patient’s satisfaction with the overall process of pain management.
Each item is scored from 0 to 1. A higher total score indicates greater satisfaction.
Scoring criteria: Dissatisfied = 0; Neutral = 0.5; Satisfied = 1;

**Questionnaire Items**

1. How satisfied are you with the level of pain relief achieved after treatment?

( ) Dissatisfied

( ) Neutral

( ) Satisfied

2. How satisfied are you with the management of breakthrough pain (sudden worsening of pain)?

( ) Dissatisfied

( ) Neutral

( ) Satisfied

3. How satisfied are you with the support provided by healthcare professionals/pharmacists in preventing and managing adverse reactions to analgesics (e.g., constipation, nausea)?

( ) Dissatisfied

( ) Neutral

( ) Satisfied

4. How satisfied are you with the clarity and adequacy of medication counseling (dosage instructions, side-effect explanations)?

( ) Dissatisfied

( ) Neutral

( ) Satisfied

5. How satisfied are you with the timeliness and usefulness of follow-up (telephone or outpatient follow-up)?

( ) Dissatisfied

( ) Neutral

( ) Satisfied

6. Overall, how satisfied are you with the service attitude and communication of the healthcare team throughout the pain management process?

( ) Dissatisfied

( ) Neutral

( ) Satisfied

**Scoring Method**

Total score = sum of all 6 items (range 0–6)
0–2 points: Low satisfaction
3–4 points: Moderate satisfaction
5–6 points: High satisfaction
