# Supplementary material for: The impact of pharmacist-led medication therapy management on the efficacy of cancer pain control: a pre-post interventional study
Source: J Pharm Health Care Sci. 2026 Jan 27;12:24. doi: 10.1186/s40780-026-00544-8 (PMC12918049; doi:10.1186/s40780-026-00544-8)
Supplement: Supplementary file 2 — Supplementary Table S2: Cancer Pain Treatment Follow-up Satisfaction Questionnaire [file 40780_2026_544_MOESM2_ESM.docx]

**Table S2 Cancer Pain Treatment Follow-up Satisfaction Questionnaire**

6 items, 3-point scale, total score 0–6

**Instructions**

This questionnaire is designed to evaluate the patient’s satisfaction with the follow-up process during cancer pain management.
Each item is scored from 0 to 1. A higher total score indicates greater satisfaction.
Scoring criteria: Dissatisfied = 0; Neutral = 0.5; Satisfied = 1;

**Questionnaire Items**

1. How satisfied are you with your pain relief during the follow-up period?

( ) Dissatisfied

( ) Neutral

( ) Satisfied

2. How satisfied are you with the timeliness of follow-up (e.g., receiving responses or support when pain worsens)?

( ) Dissatisfied

( ) Neutral

( ) Satisfied

3. How satisfied are you with the frequency of follow-up? Do you feel it adequately meets your needs for pain management?

( ) Dissatisfied

( ) Neutral

( ) Satisfied

4. How satisfied are you with the explanations provided by healthcare professionals/pharmacists regarding the use of analgesics and related precautions during follow-up?

( ) Dissatisfied

( ) Neutral

( ) Satisfied

5. How satisfied are you with the assistance provided in managing adverse reactions (e.g., constipation, nausea, drowsiness) during follow-up?

( ) Dissatisfied

( ) Neutral

( ) Satisfied

6. How satisfied are you with the communication attitude, patience, and support provided by the healthcare team during the follow-up process?

( ) Dissatisfied

( ) Neutral

( ) Satisfied

**Scoring Method**

Total score = sum of all 6 items (range 0–6)
0–2 points: Low satisfaction
3–4 points: Moderate satisfaction
5–6 points: High satisfaction
